# Supplementary material for: Comprehensive analysis of male-free reproduction in Monomorium triviale (Formicidae: Myrmicinae)
Source: PLoS One. 2021 Apr 29;16(4):e0246710. doi: 10.1371/journal.pone.0246710 (PMC8084239; doi:10.1371/journal.pone.0246710)
Supplement: Attachment — Submitted filename: Response to Reviewers.doc [file pone.0246710.s003.doc]

Dear Dr. Chaline,

We are very pleased to have your valuable comments on the manuscript entitled “Comprehensive analysis of male-free reproduction in *Monomorium triviale* (Formicidae: Myrmicinae)" (PONE-D-21-02452). Based on the important advice from the two reviewers, we have made corrections as much as possible, and the following revisions have been made. Newly added supporting information S1 File responded to comments from reviewer #2 by confirming complete worker sterility in response to. We hope this revised manuscript could now be acceptable for publication.

Sincerely,

Naoto Idogawa and Shigeto Dobata

Response to Reviewers

We appreciate your constructive advice and have incorporated them into our manuscript as much as possible, as described below. In the file derived from the original WORD file, the revised sentences are highlighted, including additional acknowledgement. Lines in your comments indicate those of the old version you have already read, and those in the revised manuscript are indicated by line numbers in the WORD file.

**Journal Requirements**

[J-1] Please ensure that your manuscript meets PLOS ONE's style requirements, including those for file naming.

I&D: We ensured that our manuscript including file naming meets PLOS ONE's style requirements.

[J-2] Please ensure you have included the full name of the authority that approved the sampling sites access and, if no permits were required, a brief statement explaining why.

I&D: We added a brief statement in the Materials & Methods (Lines 71) that no specific permission for sampling was required.

[J-3] We note that Figure 1 in your submission contain map images which may be copyrighted. (~~) We require you to either (1) present written permission from the copyright holder to publish these figures specifically under the CC BY 4.0 license, or (2) remove the figures from your submission.

I&D: In our Figure 1, we used map data from Natural Earth (public domain: <http://www.naturalearthdata.com/>). We indicate the data source credit in figure legend (line 81-82).

[J-4] Please review your reference list to ensure that it is complete and correct.

I&D: We checked the reference list as much as possible.

**Reviewer #1**

We highly appreciate your valuable comments. We made the following revision:

[1-1] Line 95: consider modifying to “First the queen was immobilized by soaking in 70% ethanol for 3 min.”

I&D: We modified the sentence accordingly (line 95).

[1-2] Line 254: should ref. 26 be ref. 27?

I&D: Thank you for your thorough reading. The citation number has been corrected (line 256).

[1-3] Line 282: consider rewording to “derived from the same daughter cell during the first meiotic division are fused to restore diploidy after the second meiotic division.”

[1-4] Similarly line 284 could be reworded to read “different daughter cells derived during the first meiotic division are fused.”

[1-5] Line 286: should read “hymenopteran” as it is used as an adjective here rather than a noun.

I&D: We appreciate your suggestion. Following comments from reviewer #2, we removed this paragraph.

[1-6] Line 312: consider replacing “habitat” with “habitats”

I&D: We modified the sentence accordingly (line 307).

[1-7] Line 318: consider replacing “Future” with “Additional” to avoid repetition since the last word of the previous sentence is “future.”

I&D: We modified the sentence accordingly (line 313).

**Reviewer #2**

We highly appreciate your constructive suggestions. We made the following revision:

[2-1] One may regret, however, that no data demonstrating worker sterility is shown or cited (i.e. dissection of workers or rearing of queenless colonies).

I&D: We added the data supporting obligate worker sterility by dissection as a supporting information.

[2-2] Besides, the discussion regarding the mode of parthenogenesis employed by *M. triviale* should be reconsidered. The authors suggest that clonal reproduction may proceed through central fusion automixis based on the observation that heterozygosity at the studied microsatellite is maintained across generations. Yet, this pattern could also result from apomictic parthenogenesis or terminal fusion automixis with selection against homozygosity.

I&D: We agree with your comment, our discussion about the mode of parthenogenesis is too speculative. Therefore, we removed this paragraph.

[2-3] There are some inconsistencies in Table 1 (there are more surviving queens than isolated queens in locality 6) and Table 4 (no locality 1 is not mentioned)

I&D: Thank you for your thorough reading. We understand that you mentioned Table 3 and 4 and corrected them.

[2-4] l76-77 and l86-88: That "ethanol-preserved samples" are only mentioned for colonies maintained alive sounds weird.

I&D: We agree with your comment. We modified the sentences (line 76-77 and 87-88).

[2-5] l300-302: One can be more precise here. Having no mutation in 639 bp of COI sequence translates to an estimated divergence time of no more than 45,000 years (assuming a divergence rate of 3.54% My−1; <https://doi.org/10.1093/molbev/msq051>).

I&D: We appreciate your suggestion: 1,000,000 * (1/639)/0.0354 ~ 45,000. We added (line 295-296) together with the relevant reference.
